# Resistance of 3D-Printed Components, Test Specimens and Products to Work under Environmental Conditions—Review

**DOI:** 10.3390/ma15176162

**Published:** 2022-09-05

**Authors:** Marcin Głowacki, Adam Mazurkiewicz, Małgorzata Słomion, Katarzyna Skórczewska

**Affiliations:** 1 Department of Mechanical Engineering, Bydgoszcz University of Sciences and Technology, Kaliskiego 7 Street, 85-789 Bydgoszcz, Poland; 2 Department of Management, Bydgoszcz University of Sciences and Technology, Kaliskiego 7 Street, 85-789 Bydgoszcz, Poland; 3 Faculty of Technology and Chemical Engineering, University of Sciences and Technology, Seminaryjna 3, Street, 85-326 Bydgoszcz, Poland

**Keywords:** 3D print, environment conditions, properties, environmental resistance

## Abstract

The development of additive manufacturing methods known as “3D printing” started in the 1980s. In these methods, spatial models are created from a semi-finished product such as a powder, filament or liquid. The model is most often created in layers, which are created from the semi-finished product, which is most often subjected to thermal treatment or using light or ultraviolet rays. The technology of additive manufacturing has both advantages and disadvantages when compared to the traditionally used methods of processing thermoplastic materials, such as, for example, injection or extrusion. The most important advantages are low cost, flexibility and speed of manufacturing of elements with different spatial shapes. From the point of view of the user of the product, the most important disadvantages are the lower mechanical properties and lower resistance to environmental factors that occur during the use of the manufactured products. The purpose of this review is to present current information and a compilation of features in the field of research on the effects of the interactions of different types of environments on the mechanical properties of 3D-manufactured thermoplastic products. Changes in the structure and mechanical properties of the material under the influence of factors such as humidity, salt, temperature, UV rays, gasoline and the environment of the human body are presented. The presented article enables the effects of environmental conditions on common materials used in 3D printing technology to be collated in one place.

## 1. Introduction

The phenomena occurring in nature are the most common inspiration for new solutions. Three-dimensional (3D) printing may seem to be an advanced technology, but many living organisms have engaging in a similar process for a long time. For example, molluscs producing their shells (calcareous exoskeleton) can be considered a natural 3D printer. As they grow, the molluscs add calcium carbonate to their external shell. In this way, more internal space is protected by the skeleton and the growth lines, similar to the layers of printed material, are visible on the outside of the shell [1].

The use of photopolymers to create 3D objects in the 20th century led to the development of fast prototyping capabilities using the Fused Deposition Modeling (FDM) technique [2,3]. Stereolithography, patented in 1986 by Charles Hull, was the first ever method for the automatic manufacturing of three-dimensional models using UV radiation and photosensitive resin [4]. At the same time, in Texas, the concept of creating objects by using a laser beam to selectively sinter powder, a technology currently known as Selective Laser Sintering (SLS), was developed [5]. Fused Deposition Modeling, or FDM, is the most commonly used method. Its applications and modifications are discussed in this review study. The method was patented in 1989 by S. Scot and Lisa Crump [6]. It involves the deposition of a polymer filament via a heated nozzle and allows rapid prototyping. The modern FDM techniques will be discussed in further detail later in the study.

Three-dimensional printing is an additive manufacturing technique, in which the product begins as a single layer applied on the base and is formed by adding subsequent layers. A large number of conventional production technologies involve subtractive manufacturing, meaning that the final product starts as a block of material, e.g., wood or metal, from which excess material is removed to form a desired shape. A large amount of waste material—for example, sawdust or metal chips—is produced in the process. Additive manufacturing techniques, such as laying a wall by adding layers of bricks and mortar according to a formal plan, have been used for many years. All the above methods, from the design to the production of the end product, have developed into modern 3D printing techniques. A key stage is the planning of the layer arrangement process, and 3D printers contain components providing automatic control [7].

Polymers are some of the most commonly used materials in additive techniques. The polymer technology is known as the “innovation of the millennium”. They are widely used because they are simple to produce and modify at a relatively low cost compared to other materials, and their wide-ranging properties can easily be adapted to many applications. The polymers are used in many domestic and industrial applications [8]. Due to a single technology, we now have the capability to create, at our own leisure, prototypes or common household items. Three-dimensional (3D) printing, or additive manufacturing (AM), is a technology widely used, not only by the industry or individuals, but also to aid scientific research. This technology allows one to create high-precision, low-cost prototypes, and, with minor hardware requirements and no special qualifications or licenses needed for the operators, it can be used by anyone. The items manufactured with this printing process are robust and lightweight, with a greatly reduced amount of waste product. AM is defined as the process of creating a 3D solid from a digital file. In this process, the building material is applied layer by layer with selective sintering [9]. Depending on the printing technology and materials used, the selective sintering process may differ. The history of 3D printing, its first uses and the available 3D printing techniques are detailed in [10,11,12,13,14,15,16,17,18,19,20].

The following techniques are available.

Fused Deposition Modeling, or FDM, uses thermoplastics for printing. A polymer, in the form of a filament, is fed into a 3D printer extruder and heated to a semi-liquid material. The first layer is applied in the *XY* axis. Next, the extruder moves up and the bed moves down by the thickness of the first layer and the second layer is applied. Hot semi-liquid polymer cools down rapidly and solidifies, forming a designed shape. The solidification rate depends on the position, glass transition temperature, flow temperature and filament thickness [10]. A detailed review of this technology can be found in many publications [19,21,22,23,24,25,26,27,28,29,30].

Stereolithography, or SLA, is a printing technique using light-cured resin (cured by a laser beam). A container is filled with light-cured resin, and, during printing, the bed is immersed in the resin to the very bottom. The laser beam traces the shape of the object in the XY plane on the bed, curing the first layer of the resin. After tracing, the bed moves up, and the process is repeated. The disadvantage of this AM technique is the small printing area, and its advantage is that the printed item is not affected by external factors [10]. A detailed description of the method and its applications can be found in [31,32,33,34,35].

Digital Light Processing, or DLP, is a technique similar to SLA, involving the curing of light-sensitive materials using light emitted by a projector. The difference is that DLP creates models by curing the whole layer at one time, and in SLA, the laser beam moves from point to point, tracing the geometry. This technology is used in many areas [36,37,38].

Jetting Molding, or MjP, is a molding technique using a process similar to a standard jet or laser printing on a piece of paper. The head moves over the table in the *X* axis and applies a light-cured resin, which in turn is cured using UV light. After printing the layer, the table moves down in the *Z* axis. Example applications of the technology can be found in [39,40,41].

Selective Laser Sintering, or SLS, uses a powdered thermosetting polymer. The applied powder layer is selectively sintered at a high temperature using a laser beam. Similar to CJP or Binder Jetting, the printed models are removed from a block of non-sintered powder [40,42,43,44].

The above descriptions of selected additive manufacturing techniques present the scale and wide range of applications of 3D printing techniques. The aim was to show the different additive manufacturing methods and the available directions of research to verify the resistance of printed models to environmental conditions.

Fused Deposition Modeling (FDM) is the most commonly mentioned technique in the literature, and the main scope of this study is based on products manufactured using this technique. The Fused Deposition Modeling (FDM) technique was developed in 1988, and the patent protection expired in 2009. It allowed different companies to design, develop and commercialize the technology in a wide range of specifications and components, including domestic, office and industrial applications. The interest in this technique peaked in recent years, particularly in medical applications, but also in the scientific field, which is clearly reflected in the many research studies on this topic [10]. The print parameters are usually recommended by the filament manufacturers, allowing less qualified operators to produce good-quality prints. This makes the technology an excellent solution in many fields. Its many advantages, including its versatility and the ability to adapt it to new materials, allow it to create multi-colored and complex end products at low costs and high speeds, and make the technique very popular. In recent years, it has found its place in medical applications, electronics and various production processes. Thanks to their many interesting properties, including low weight, high rigidity and low density, the layered structures are widely used in many fields of industry [40].

The initial process conditions selected at the planning stage will affect the quality and structure of the product. No information can be found in the literature on how the environment affects the prints using different materials. There are many studies that research the effects of different parameters on the quality and mechanical properties of prints, but no systemic information can be found in the literature on how the prints are affected by environmental factors. The FDM prints can be exposed to many environmental factors that may affect their strength parameters in time.

Depending on the filament and modifiers (fillers, dyes, pigments, etc.) used, many different prints can be produced with different properties depending on the consumer requirements. Filament materials including acrylonitrile butadiene styrene (ABS), acrylonitrile styrene acrylate (ASA), polylactic acid (PLA), polyethylene terephthalate glycol (PETG) or polyamide (PA) are commonly used in different AM processes. The filament used and its modifiers can significantly affect the resistance of the prints to environmental factors.

The properties of 3D prints are determined based on the analysis of basic mechanical properties, including bending, compression, tension or impact. A surface structure is also assessed using scanning electron microscopy (SEM) or computed microtomography (CT) to detect any defects and analyze the structure of materials produced in the AM processes. The testing of additive techniques is also based on the analysis of mechanical properties. Although the polymers are widely used in 3D printing, a small number of researchers deal with the effects of environmental conditions with regard to a specific set of conditions.

**Figure 1:**
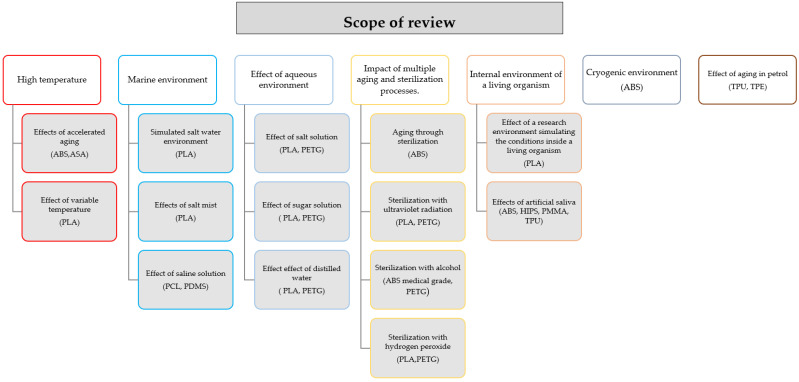
The range of subjects discussed in the review, along with the materials.

The present article includes a current review of the research on the resistance of polymer 3D prints to different environmental factors. The order of the discussed environments with materials is shown in Figure 1. It will allow a systematic review for a better understanding of the effects of environmental conditions on the structure and durability of the prints. It will also allow us to determine further directions of research conducted on this topic. The knowledge will allow us to extend the applicability of the products obtained using AM techniques depending on the material used and its exposure to environmental factors.

## 2. Effect of Thermal Aging Conditions on the Properties of 3D-Printed Samples

One of the most commonly considered effects of the environment on the print quality is the effect of temperature on the properties of components subject to thermal aging. M. Reza Khosravani et al. [44] analyze the effects of accelerated aging on 3D prints. The products, made of ABS and ASA with a hexagonal and triangular fiber arrangement, were printed with 30% infill density and no outer layer, to allow the analysis of the core structure and its structural evaluation. To prevent defects, including gaps, overlaps or voids, at the production stage, a surface layer made of an epidermal material—a carbon fiber composite—was added. The thermal aging procedure involved placing the test specimens in a climatic chamber at temperatures below the glass transition temperature of the tested material (between 22 °C and 60 °C). The test lasted 240 h. A change in test specimen mass before and after thermal aging was determined. Tensile, bend and compression tests were also carried out [45].

The test specimens, under thermal aging, showed improved rigidity and durability, and the change in mass and dimensions after the aging process was approximately 1%. Despite the 1 wt.%. change in mass, the bending stress in the aged test specimens increased by approximately 15%, which may have been due to annealing as a result of aging, which in turn led to an intermolecular rearrangement [45]. The structure of the aged ASA and ABS components changed, increasing the maximum bending stress, modulus of elasticity and bending resistance. The temperature caused an increase in maximum durability for both fiber arrangements and materials by changing the molecular structure. The ASA component with a honeycomb structure showed the highest breaking strength. The hexagonal core arrangement was the best for both materials, since it resulted in higher strength of both components. The authors of the study highlighted the effect of the working temperature and the core structure as the key parameters in designing components made of ABS and ASA. During the design of the components using 3D techniques, the printing conditions and the printing process must be adapted to the thermal working conditions of the component [45].

The topic of the effect of temperature on the mechanical properties of PLA was discussed by Y. C Niranjan et al. [45]. The authors considered the effects of annealing parameters on the dynamic mechanical properties of PLC components printed using the FDM technique. Six test specimens, 50 mm × 11 mm × 3 mm, were printed and annealed in a convection oven. The test specimens were divided into two groups. The first group of the test specimens was characterized by a variable holding time in the oven at 90 °C. The effect of the holding time (15, 30 and 60 min) on the PLA test specimens at a constant temperature regarding the dynamic mechanical properties was analyzed. The second group of the test specimens was heated for 30 min at different temperatures, namely 80 °C, 90 °C, 100 °C and 110 °C. The PLA heating temperature was limited to 110 °C, since, over 120 °C, the material had deteriorated visibly, affecting its mechanical properties and geometric features. After cooling to room temperature, the test specimens were stored at room temperature for two days to simulate the actual working conditions, followed by dynamic mechanical analysis (DMA).

The results show that both the annealing duration and temperature significantly affect the dynamic mechanical properties of PLA printed using the FDM technique. The heat increased the conservative modulus, the resistance to bending of the PLA components and the glass transition temperature of the tested material. The loss modulus of the annealed test specimens tended to decrease, both at room and increased temperature, due to the increased rigidity of the annealed PLA components compared to the base material. The results showed that the annealing process can also contribute to bonding strength between the layers, and the reduced void content and increased crystallinity can have a positive effect on the stiffness of the viscoelastic polymer.

Annealing processes can be used as a low-cost process to improve the mechanical properties of the material. The interesting effects of thermal aging on the components produced using additive techniques can be found in [46,47,48,49,50,51,52,53,54,55,56,57,58,59,60,61].

## 3. Susceptibility of 3D Prints to Simulated Marine Environment Conditions

Studies discussing the effects of marine environments [62], salt mist [63] and saline solution [64] are the main sources of information on the effects of environmental factors on the mechanical properties of polymer 3D prints. A study discussing the effects of biofouling on 3D-printed components made of PCL immersed in salt water is also available [65].

R. Krishna Upadhyay et al. [62] described a test consisting of immersing the test specimens for 30 days in a simulated salt water environment. The material used for printing was polylactic acid (PLA). The test specimens of different shapes were made using additive manufacturing techniques. These included both standard test specimens for strength testing, propeller blades and plates for the evaluation of the effects of salt water on the material surface structure.

The changes occurring as a result of print storage in a simulated marine environment to ASTM D1141-98 were evaluated. Friction, wear and sliding wear mechanisms, mechanical properties (tensile strength and Vickers hardness) and the surface roughness of the test specimens were analyzed. To determine the changes occurring in the test specimen structure after 30 days in salt water, the test specimens were analyzed using a scanning electron microscope (SEM).

The results of the experiment showed a change in the mechanical properties of the PLA test specimens. The surface roughness measured on both surfaces of the test specimen was much higher than before, which may have an effect on their intended use. After submersion in salt mist for 30 days, the physical properties of the test specimens had changed. The liquid particles that migrate inside the rough structure may degrade the polymer more effectively as a result of a hydrolytic reaction. Low surface roughness significantly affects the tribological properties and ensures problem-free operation and higher resistance to hydrolytic degradation.

The tensile test was conducted at a high load (25 mm/min) to simulate the use of the material in an environment with theoretical conditions present in its actual application. The analysis of the mechanical properties of the primary test specimens and test specimens subjected to the salt mist concluded that the marine environment reduced the PLA test specimens’ elongation at rupture, while increasing the yield point and the modulus of elasticity. The analysis of the mechanical properties shows that 3D-printed components can be used for target marine applications, including slide-bearing operations at moderate speed and load.

It is worth noting that the analysis of the external structure of the turbines in [66] after exposure to salt mist showed a deterioration in their surface; however, the observed changes were minor. The XRD spectra of the test specimens before and after salt mist exposure were identical, and the minor changes in 2theta angle within 20–27 2theta indicate the amorphic nature of the PLA used to produce the test specimens. In summary, the 3D-printed PLA components showed satisfactory mechanical strength and the marine environment had a low effect on material deterioration in the analyzed period. This means that the 3D components can be used in slide applications at moderate loads in the analyzed environment.

S. Ambrus et al. presented, in [63], the test results of the analysis of a material aged in salt mist. The components were produced from PLA using the FDM technique. The component production parameters and the crystallinity parameters of the produced components were determined using an XRD technique.

The study was carried out in accordance with the guidelines for component testing in an artificial atmosphere [63]. A Liebisch SI-400 device was used for observation of the mechanical behavior of the test specimens after being held in specific conditions in the experiment involving aging in salt mist. The test specimens were subjected to moisture–thermal testing at 45 °C, with subsequent rinsing with distilled water and 3.5% NaCl solution at a 0.8 m^3^s^−1^ flow rate. The test lasted 240 h.

The breaking strength of the test specimens before the aging process ranged from 44.27 MPa to 39.75 MPa, with an average value of 42.14 MPa. Following 240 h testing in sprayed salt mist conditions, the breaking strength of the test specimens showed higher variability, i.e., between 31 MPa and 40 MPa, with the tensile stress ranging from 31 to 34 MPa. The impact test did not yield any significant differences.

The study concluded that no change in test specimen mass was observed, although the strength decreased due to polymer degradation as a result of component aging in the salt chamber.

A non-typical study on the effect of biofouling, i.e., formation of a biofilm as a result of biological contamination in sea water, leading to the biodegradation of plastics, was discussed in the article by Matthew Ryley et. al. [66]. The mechanical degradation of PCL and PDMS glass plates immersed in water was analyzed. The plates were immersed in ultrapure water for 120 h; the water was replaced after the first 24 h to remove potential eluates. The test specimens were rinsed with 70% ethanol solution and immersed in ultrapure water. The printed plates were placed for 12 weeks under a raft (a suspended weight) to prevent them from rising to the surface, at a depth of 6 meters. In this period, the sea water quality parameters, including temperature, conductivity, salinity, pH and dissolved oxygen, were monitored.

After this, an analysis was carried out to determine the differences between the control sample and the aged material. To determine the relationship between the percentage of microfouling coverage and the material properties, statistical analysis (ANOVA or Tukey post hoc tests) was carried out. In the first 4 weeks, in salt water, lower coverage by cyanobacteria was observed on PCL test specimens compared to other materials, and after 12 weeks, all materials were severely fouled by the microorganisms.

PCL was considered the best material due to the lowest overall coverage by microalgae, which, after 12 weeks, was between 86.8% and 98.4%. Among the compared materials, PCL was the least hydrophobic and showed the lowest biofilm coverage of all materials, making it the best choice for marine applications. It is also biodegradable in the marine environment. Moreover, 3D-printed polymers meet the conditions for marine applications; however, they must also be analyzed for degradation by fragmentation, hydrolysis or biodegradation as a result of salt water or microorganisms [66]. The topic was also discussed in [65,67,68,69,70,71].

## 4. Effect of Aqueous Environment, Salt Solution and Sugar Solution

D. Moreno Nieto et al. [72] presented the results of changes in PLA and PETG properties as a result of interaction with different aqueous environments. The main reason for addressing this issue was to verify the strength of materials used as packaging in the food industry. The test specimens printed for the purpose of the experiment were square-shaped, 30 mm × 30 mm × 3 mm. Sets of 15 test specimens were placed in distilled water and saturated salt and sugar solutions. The test specimens were immersed in metal containers and secured against movement using tin wire. The containers were stored at a constant temperature of 20 °C and 50% humidity. The solution was stirred once a day and replaced once a week.

The experiment involving the degradation tests lasted 10 weeks, whereas the absorption tests were continued until the test specimen mass had stabilized. The measurements were carried out every week at the same time, and if degradation was observed, the absorption was monitored every day for the first week of observation. The method of assessing the degradation involved a weekly sampling of 5 out of 15 test specimens immersed at the beginning of the test, and removing the layer of salt or sugar crystals adhered to their surfaces. The test specimens were dried on absorbent paper and stored in plastic bags. The tests included weighing, measuring and observation under an optical microscope. After this, the samples were immersed in the respective solutions again. The analysis of the experiment’s results showed that the test specimens immersed in saturated salt or sugar solutions did not show any changes in color or shape compared to the test specimens immersed in distilled water only.

The absorption tests for the PLA and PETG test specimens were conducted for 10 weeks. Visible absorption was observed in the initial phase of the experiment, i.e., in the first two or three days. An increase in test specimen mass and equilibrium lasting until the end of the experiment was observed. The swelling of the PLA samples had ceased after 8 weeks in the distilled water, 9 weeks in the sugar solution and 3 weeks in the salt solution. For the PETG sample, the equilibrium of swelling in the distilled water was observed after 8 weeks, in the sugar solution after 9 weeks and in the salt solution after 7 weeks.

The degradation effect of these environments was also evaluated. The degradation changes were determined using optical microscopy. The changes occurring in the test specimens and crystalline structures were observed in all three solutions. The observed degradation changes depended on the duration of the experiment. The changes were characterized by dark inclusions and discoloration of the PETG test specimens. Changes in the color of the PLA test specimens were also observed.

To summarize, PETG was the most dimensionally stable among the three solutions, and in a 9-week period, the change in mass was observed to be 0.3%, making it the best option for marine applications. After 8 weeks, the PLA specimens changed in mass by approximately 2.5%.

## 5. Effect of Multiple Aging and Sterilization Processes

The number of studies on polymers discussing the effects of different types of sterilization procedures has recently increased due to the lack of first aid equipment as a result of limited deliveries, further affected by the COVID-19 pandemic. In [73], Diana Popescu et al. presented an analysis of the effects of multiple sterilization procedures on polymer components and their properties. Catalin Gheorghe Amza [64] analyzed the effect of ultraviolet radiation on test specimen aging. Krzysztof Grzelak et al. [74] discussed the effect of chemical disinfection using alcohol, and in [75], Olivier Oth et al. described the low-temperature sterilization methods using hydrogen peroxide.

The first mentioned article [73] discussed the effects of the process used in a hospital environment to neutralize potential pathogens on medical instruments. The analysis covered ABS. The aim of the experiment was to evaluate the effect of each analyzed factor, i.e., aging only and aging with sterilization, on mechanical properties and structures. This process involved cyclic tests carried out during the first week of exposure to analyzed factors. The tests were carried out for 9 weeks. Mechanical tests, including bending and tensile strength, were also carried out. The test specimens had a 100% infill density and a vertical orientation, which significantly reduced the strength during strength testing due to the opposite distribution of the fibers in relation to the forces acting on them.

The test specimens were sterilized every two weeks, and the process involved exposure to hydrogen peroxide vapors and low-temperature gas plasma. Sterrad equipment was used to expose the test specimens to 45 min sterilization cycles at 134 °C and 0.223 MPa. For the first group of the test specimens, SEM image analysis showed plastic cracking of fibers and delamination between the layers of printed materials that were subjected to identical aging and sterilization conditions. Cracking, with small amounts of material being separated as a result of exfoliation, was observed for the first group aged in the storage process. Cracking along the layer printing direction in both groups was observed at the end of the experiment. Following a tensile test, an analysis of the external structure was carried out for selected series using a micro-CT instrument. The results showed that the ratio of voids to the total volume of the analyzed test specimens was between 6.14% and 7.82% due to the effects of aging and the exfoliation of printed layers.

To summarize, the test specimens were compared at selected intervals, evaluating both their mechanical strength, external and internal structure and change in mass. The results showed no significant changes in mechanical properties regarding rigidity, mass loss and tensile and bending strength, irrespective of the aging technique used.

Catalin Gheorghe Amza et al. [64] discussed topics related to the effects of accelerated aging by exposing 3D-printed test specimens to ultraviolet radiation. The test involved an experimental simulation of the effects of atmospheric conditions and sun on components made of polymers, including PLA and PETG, by irradiation with a UV-A light in a radiation chamber according to ISO 4892-3:2016 [76].

In total, 64 test specimens, in the shape of a dog bone and a 15 mm × 15 mm cube, were printed for the purpose of the test. The test specimens were divided into a refence group and a test group and exposed to 310 nm wavelength radiation. A thermostatic climatic chamber, the Discovery DY110C, was used to experimentally determine the effect of UV radiation. Total UV exposure time was 24 h and included three irradiation and condensation cycles. Each irradiation cycle included an 8 h holding period at 50 °C and 50% humidity with 0.43 mW^−2^ × nm^−1^ lamps on. The alternating cycle without irradiation included a 4 h rest time with UV lamps off. The total exposure of the material to UV radiation in the chamber corresponded to several months of exposure to the sun in external conditions [64].

The visual inspection showed certain changes in color; however, the dimensional analysis did not show any effect of accelerated aging on changes in the analyzed properties of the components. The analysis of the strength tests (tensile, compression and rigidity) showed a 5.3% decrease in the strength of the PLA test specimens exposed to UV-B radiation. The test specimens were more fragile, losing 6.3% of their compression strength, whereas the rigidity of the irradiated test specimens did not change significantly. For PETG test specimens, a significant 36% strength decrease compared to the control test specimen was observed. Compression strength decreased by 38.6%, whereas the rigidity did not change. The cracking analysis using the SEM technique showed changes in roughness at the surfaces of fibers, which may indicate a degradation process [64].

In PLA test specimens exposed to UV-B radiation, the mechanical strength decreased slightly, whereas significant changes were observed for the PETG test specimens subjected to identical exposure conditions. The analysis of the test results, particularly the mechanical properties of 3D prints, showed a detrimental effect of UV radiation on the components intended for use in direct sunlight [64].

Krzysztof Grzelak et al. analyzed the effects of another sterilization method—alcohol disinfection—on the print properties [74]. The tests included three materials, i.e., PETG filament with color pigment and without additives and ABS for medical purposes, which is a dedicated material for medical applications. The reason for choosing this disinfectant was that it is readily available in most healthcare facilities.

Five test specimens of each material (in the shape of a dog bone) were printed for the purpose of this test. The test specimens were divided into four groups with different exposure times, as well as a control group. Each group was characterized by a different disinfection time, i.e., 0.5 h, 12 h, 24 h and 48 h, respectively. To determine the effect of the disinfectant concentration, selected test specimens were placed in a disinfection container filled with 4% aqueous alcohol solution and selected test specimens were placed in an undiluted disinfectant. After a set disinfection time, the components were dried in a laboratory drier for one hour at 45 °C.

The results showed a minor effect of disinfection on the material structure and its tensile elongation and change in mechanical properties irrespective of the exposure time to disinfectant in the analyzed 48 h period. The slight impact may have been caused by the penetration of liquid molecules into the structure of the samples. The analysis showed that the disinfectant had a minor effect on the PETG prints. Moreover, the type of dye used did not show any effect on the mechanical properties of the material.

Similar effects were observed during polymer tests with different disinfection techniques, e.g., photodynamic [77] or microwave [78]. Among the two types of analyzed materials, the dedicated material—medical-grade ABS—seemed to be a better candidate for component production using AM techniques.

Non-modified PETG (without additives) is at risk of a decrease in tensile strength by 20%, and the addition of a dye slightly increases the chemical resistance of the material [74].

One of the mentioned methods to destroy microorganisms is low-temperature sterilization with hydrogen peroxide. The effect of this technique was analyzed for surgical guides produced using the AM technique. The components described in [75] were designed at the maxillofacial department and made of PETG or PLA. The materials were sterilized in a single 50 min cycle at a temperature of below 55 °C.

Both before and after sterilization, the guides were scanned using a CT scanner and the deviation of starting points of the geometry measurement was compared to the reference guide. A statistical analysis, an ANOVA test with random factors, allowed the authors to determine the morphometric differences between different guides. For PLA, the high temperature used in the conventional sterilization techniques (121 °C) resulted in the material melting after a short cycle (5 min), which was verified by the authors with simultaneous discussion of the results obtained by Boursier et al. [79].

The results of the sterilization with hydrogen peroxide unequivocally showed no effects on the material structure. The components made from these materials can be used for clinical applications. The morphological differences were less than 0.2 mm. The analyzed method can be used as an alternative method to avoid the deformation of 3D prints made of PLA and PETG during sterilization. The results of tests describing the importance and the effects of sterilization are included in [80,81,82,83,84,85].

## 6. Internal Environment of a Living Organism

Amirapasha Moetazedian et al. [86] attempted to analyze the effect of a research environment simulating the conditions inside a living organism on the tensile strength of polylactic acid (PLA) components produced using AM techniques. To determine the consequences of placing a plastic implant inside a living organism, the factors present inside the organism must be simulated. The study aimed to determine the effects of three different conditions on the mechanical properties of PLA prints. The effect of an aqueous environment (immersion and increased humidity) and the effect of temperature (room and body temperature (37 °C)) were determined.

To check the component’s saturation with water, the absorption was tested within 48 h. At room temperature, after 30 min, the absorption reached 0.561% and did not change until the end of the test. An increase in water temperature increased the absorption to 0.741%. After determining the change in mass, the tensile strength was tested. For the test specimens stored at room temperature, the tensile strength decrease was 0.188%; however, for the second type of test specimens exposed to a physiological environment, a significant weakening of the polymer by 23.4% relative to the reference test specimen was observed. Water absorption by the test specimens before the tensile test significantly affected the elongation at break, whereas changes in the yield point were minor. PLA components fully immersed in water showed a significant change in key properties relative to the control test specimens.

A synergistic effect of high temperature and water absorption resulted in a transition from brittle cracking to intermediate brittle cracking. A significant decrease in the mechanical parameters of 3D-printed test specimens was observed. Immersion in water at 37 °C resulted in a 50% reduction in the mechanical strength of the polymer and a 20% reduction in the tensile modulus of elasticity. The test specimens were deformed at 40% of the reference test specimen.

The test results do not reflect all synergistic environmental conditions present in living organisms due to the lack of use of biomedical materials, i.e., fluids corresponding to those present in the human body.

The second article discussing issues related to the effect of a physiological environment on 3D-printed polymer components was written by Any C. Pinho et al. [87] and describes the effects of artificial saliva on multi-material prints. The experiment covered the properties of test specimens made of a single material or two materials, where TPU was the core and the outer layer used materials including ABS, HIPS and PMMA. The core material was selected based on its ability to disperse impact energy. For each type of configuration and mechanical test, 12 test specimens with 100% infill were printed.

Half of the printed test specimens of each material were aged in artificial saliva before the mechanical tests. The artificial saliva was made by adding 0.426 g (Na_2_HPO_4_) disodium hydrogen phosphate, 1.68 g (NaHCO_3_) sodium bicarbonate, 0.147 g (CaCl_2_) calcium chloride and 2.5 mL (HCl) hydrochloric acid to 800 mL distilled water. Single test specimens were placed in tubes with the prepared solution and left in a shaker for 14 days at a constant temperature of 37 °C at 100 rpm. The procedure aimed to simulate the effect of exposure to saliva on the test specimens for one year.

The test results did not show any significant changes in the dimensions of the components immersed in saliva, which confirmed that the analyzed thermoplastic polymers showed low affinity to aqueous solutions. The same was validated by the results of studies published in [88,89]. The results of the mechanical property tests showed similar trends both for dry test specimens and aged test specimens, except for the ABS and PMMA test specimens, where the maximum bending stress was lower in the case of the aged test specimens. The opposite trend was observed for the HIPS and TPU test specimens.

The information showed that the aging process in a simulated saliva environment did not significantly affect the mechanical behavior of the test specimens, except for the PMMA, which is the only polymer without any aromatic rings in its chemical structure, which may explain its different behavior. The ABS test specimens lost 28.7% elasticity and 34.4% of absorbed energy as a result of the aging processes in the saliva solution.

For the sandwich test specimens, the behavior of all layered structures changed slightly as a result of aging in artificial saliva. Using TPU as a core affected the reduction in the mechanical properties of multi-material test specimens. The highest decrease in strength was observed in the test specimens containing PMMA, probably as a result of lower adhesion between the polymer and TPU, resulting from the difference in chemical composition.

To summarize, the saliva affected some of the multi-material prints. The combination of TPU with other materials affected the bending strength of the test specimens. Among all the material combinations, the best one was ABS-TPU-ABS due to the highest elasticity of the test specimens [87]. The effect of high moisture and variable temperature was discussed in [90,91,92,93,94,95].

## 7. Cryogenic Environment

Increased temperature is one of the factors affecting polymers during production or use. In [96], F. Saenz et al. tested the mechanical properties of ABS components produced in additive manufacturing processes, both at room temperature and at −196.15 °C [97]. The manufactured parts were HTS magnetic coils used at low temperatures, expected to transfer moderate mechanical loads at the supports [96]. The effects of cryogenic temperatures on polymers are widely discussed in the literature, and the results may validate the applicability of ABS in the analyzed applications.

For the purpose of an experiment, a cryogenic tank was designed and constructed for use with liquid nitrogen. The test specimens for mechanical tests (32 test specimens) were stored in the cryogenic tank. The test specimens, 115 mm × 19 mm × 6 mm, in the shape of a dog bone, were made with 80–90% infill. The cooling procedure involved placing the test specimen inside the cryogenic container. The cooling time was 3 min due to the insulation added to the test specimens.

Testing of the mechanical properties, both the yield point, Young’s modulus and dry strength of the test specimens stored at −196.15 °C, did not show any significant differences for all tensile tests. The maximum strength was reduced by 4–7%, which is a typical effect of material brittleness at low temperatures. For comparison, the strain range for the test specimens stored at room temperature was between 15% and 28%.

The strength tests for test specimens stored at 77 K showed elastic behavior until breaking, a higher Young’s modulus and a lower strain rate compared to the test specimens stored at room temperature. A statistical analysis was used to determine the significance of the results. The results showed that the brittleness of ABS test specimens produced using additive techniques increased with a decrease in temperature to 77 K. Based on the analysis of the results, the authors found that the infill density and the pattern affected the change in yield point, Young’s modulus and dry strength. The effect of negative/cryogenic temperatures was discussed in [98,99].

## 8. Effect of Aging in Petrol

Eva Paz et al. [100] discussed issues related to the effect of an abrasive medium—in this case, petrol—on test specimens produced using additive techniques and made of two different materials, TPU and TPE. The study allowed them to determine the effects of a chemical abrasive medium on changes in mechanical properties in time.

The components for the purpose of the study were printed with 5%, 20%, 50% and 80% infill and a rectangular infill pattern. Five test specimens for each infill density, test type and material aging time were printed. The exposure to chemical factors was measured in accordance with ISO 175:2010 regarding the testing of plastics in liquid chemicals [101]. The samples were stored in containers filled with 98 octane petrol, until the samples were fully immersed. The material was stored in a chamber with temperature control and an air exhaust to avoid the possible accumulation of petrol vapors. The test periods were 24 h and 7 days, corresponding to a short-term and long-term test according to the standard. The test specimens were also exposed to petrol for 30 days.

The results for both materials, before and after aging, were compared. TPU components showed higher strength compared to TPE, irrespective of the external factors. For test specimens with over 20% infill density, no significant effects on material hardness were observed at different periods of immersion in petrol. As a result of aging, the TPE test specimen mass increased due to petrol absorption, compared to the TPU test specimens, showing that the TPE test specimens had lower resistance to petrol compared to the TPU test specimens. The test showed that the infill density had a significant effect due to the decrease in petrol absorption rate, resulting in a minimal effect on mechanical properties. The samples with 80% infill density showed surface-only interaction with the solvent.

The results suggest a two-stage interaction between the polymers and the analyzed environment. The first resulted in the breakage of some of the chains forming the polymer, resulting in a loss of mass and the deterioration of its properties. The second resulted in the recovery of the original properties due to the evaporation of the absorbed solvent from the intermolecular lattice of the polymer. For TPE, this process takes less time to complete.

## 9. Conclusions

The availability and easy application of additive technologies make them popular in many applications. The ease of manufacturing a specific product adapted to individual client needs means that the materials are widely used in many applications. Due to their applications, the components may be exposed to different environmental factors, the effects of which are not fully classified or well known. Another factor affecting the changes in properties is the printing technique used and the choice of materials.

In the discussed experiments involving exposure to different environmental factors, the properties of the components printed using materials most commonly used in additive technologies, including ABS, ASA, PLA, PETG, HIPS, TPU, TPE, PMMA or PCL, were analyzed. The components that can be used as spare parts in many devices were exposed to selected controlled environments, including disinfectants, low and high temperatures, humidity, different liquids and aqueous environments.

The analysis of the results of accelerated aging based on dynamic mechanical characteristics showed that both the annealing time and the temperature caused an increase in the conservative modulus for PLA, resulting in improved mechanical properties of the material [45]. It is worth emphasizing that the infill density is of key significance due to the absorption rate of the medium. The tested specimens with 80% infill density showed surface changes only [10].

PLA test specimens exposed to a simulated marine environment by immersion in salt water and PLC test specimens exposed to salt mist showed only a minor deterioration in the external structure [62,63]. The results do not exclude use of components made of polymers manufactured using additive techniques in the analyzed environment.

The effects of an aqueous environment and a salt and sugar solution on the properties of PLA and PETG prints were evaluated, including absorption and external structure analysis. A change in color in the test specimens immersed in distilled water was the most clear, and the lowest absorption rate and mass stability were observed for PETG test specimens [72].

Sterilization, both using hydrogen peroxide vapors [73] to disinfect ABS, alcohol to disinfect PETG [74] and ultraviolet radiation [75], did not show a significant effect on ABS, PLA and PETG test specimens. The first sterilization method did not affect the component properties, the second showed a minor effect on the material structure, and the third method showed a minimal effect on the mechanical properties of PETG samples only.

A simulation of the internal environment of a living organism was described in [86]. In the mentioned study [86], the environment was simulated without any biomedical fluids, which prevented the simulation of all synergetic conditions present in a living organism. An increase in mechanical properties and 40% deformation compared to the reference sample were observed for PLA test specimens. Another study [87] analyzed the effects of artificial saliva on the properties of multi-material prints made of TPU, ABS, HIPS and PMMA. The chemical structure of PMMA significantly affected the mechanical properties due to the lack of aromatic groups in the macromolecule and thus lower chemical resistance.

Short-term storage of test specimens at −196.15 °C was used to simulate a cryogenic environment and did not show a significant effect on the mechanical properties of ABS test specimens. A reduced maximum strain was observed, which is a phenomenon typical of materials at low temperatures [96]. The following Table 1 presents a summary of the environments along with the research methods considered.

The results, presented in a collective manner, make it possible to discern similarities in terms of the evaluation of the structure or mechanical properties of polymeric materials. The most frequently selected materials for testing were, in the following order, ABS, PLA and PETG. A research gap for further studies may be to investigate the effects of exposure environments on an even wider range of materials and possible strategies to assess changes in mechanical properties—in particular, cyclic fatigue tests.

In the presented review of the literature, components produced using AM techniques were exposed to different environmental factors. The review showed that there is only a small amount of information showing a synergistic effect of different variable environmental factors on the changes in properties of 3D prints; thus, the authors plan to undertake further research on this topic. Experiments should be systematized in terms of the utility properties evaluated, as there are a large number of unknowns and it is impossible to compare relationships. Observation of the behavior of materials exposed to different factors has allowed researchers to draw conclusions on the range of changes in surface structure and variations in strength ranges depending on the 3D printing technique used.

## 10. Predictions for Further Research

There are also other factors that affect the resistance of 3D-printed elements, which are not described in the paper. Our review may be supplemented with a description of the following issues: the mechanism of damage to the structure of elements produced in 3D technology under the influence of the environment; the effects on the resistance of the parameters of the 3D printer settings; the impact of the modification of the material by adding additional ingredients, e.g., fibers or powders; the influence of the shape of the internal structure of elements resembling natural composites such as bone or wood; and the ways in which post-processing, e.g., annealing or acetone vapor, increases the resistance of components in the work environment [102,103,104,105,106,107,108,109,110,111,112,113].

All these issues have an impact on the resistance of 3D-printed elements in the work environment. However, it is impossible to discuss them in detail within one work, due to the large scope of each topic. Therefore, we recommend that these topics are considered in future papers.

## Figures and Tables

**Table 1:** Influence of the discussed environments on specific material properties.

	High Temperature	Marine Environment	Aging through Sterilization	Sterilization with Ultraviolet Radiation	Sterilization with Alcohol	Sterilization with Hydrogen Peroxide	Internal Environment of a Living Organism	Cryogenic Environment	Effect of Aqueous Environment, Salt Solution and Sugar Solution	Aging in Gasoline
**Static bending**	ABS ↑ [44] ASA ↑ [44] PLA ↑ [45]		ABS ⟷ [73]				ABS ↓ [87] PMMA ↓ [87] HIPS ↑ [87] TPU ↑ [87]			
**Tensile strength**		PLA ↑ [62] PLA ↓ [63]	ABS ⟷ [73]	PLA ↓ [64] PETG ↓ [64]	PETG ↓ [74] ABS (medical grade) ⟷ [74]		PLA ↓ [86]	ABS ↓ [96]		TPE ↓ [100] TPU ↓ [100]
**Impact strength**		PLA ⟷ [63]					ABS ↓ [87] PMMA ↑ [87] HIPS ↑ [87] TPU ↓ [87]			
**Rigidity**				PLA ↓ [64] PETG ↓ [64]						
**Dynamic mechanical analysis**	PLA ↑ [45]									
**Compression**				PLA ↓ [64] PETG ↓ [64]						
**Biofilm coverage**		PCL ↑ [66] PDMS ↓ [66]								
**Degradation changes**						PLA ⟷ [79] PETG ⟷ [79]			PETG ↑ [72] PLA ↓ [72]	

↑ Increase in strength values. ↓ Decrease in strength values. ⟷ No impact.

## Data Availability

The data presented in this study are available on request from the corresponding author.

## Nomenclature

ABS	Acrylonitrile Butadiene Styrene
AM	Additive Manufacturing
ASA	Acrylonitrile Styrene Acrylate
CT	Computed Microtomography
DMA	Dynamic Mechanical Analysis
FDM	Fused Deposition Modeling
HIPS	High-Impact Polystyrene
PA	Polyamide
PCL	Polycaprolactone
PDMS	Polydimethylsiloxane
PETG	Polyethylene Terephthalate Glycol
PLA	Poly Lactic Acid
PMMA	Poly(methyl methacrylate)
SEM	Scanning Electron Microscopy
Tg	Glass Transition Temperature (°C)
TPE	Thermoplastic Elastomer
TPU	Thermoplastic Polyurethane
UV	Ultraviolet
